# First karyotype description of *Hypostomus iheringii* (Regan, 1908): a case of heterochromatic polymorphism

**DOI:** 10.3897/CompCytogen.v6i2.2595

**Published:** 2012-03-16

**Authors:** Josiane Baccarin Traldi, Marcelo Ricardo Vicari, Daniel Rodrigues Blanco, Juliana de Fátima Martinez, Roberto Ferreira Artoni, Orlando Moreira-Filho

**Affiliations:** 1 Universidade Federal de São Carlos, Departamento de Genética e Evolução, Rodovia Washington Luís Km 235, São Carlos-SP, 13565-905, Brazil; 2 Universidade Estadual de Ponta Grossa, Departamento de Biologia Estrutural, Molecular e Genética, Av. Carlos Cavalcanti, 4748, Ponta Grossa-PR, 84030-900, Brazil

**Keywords:** chromosome variation, fish, heterochromatinization, Hypostomini

## Abstract

In this study, which is the first karyotype analysis of *Hypostomus iheringii*, nine specimens collected in Córrego da Lapa (tributary of the Passa-Cinco River) showed a diploid number of 80 chromosomes. Silver nitrate staining and fluorescence *in situ* hybridization (FISH) with an 18S rDNA probe revealed the presence of multiple nucleolus organizer regions (NORs) (chromosome pairs 13, 20, and 34). FISH with a 5S rDNA probe showed that this cistron was only present in chromosome pair 2. When the karyotypes of individual animals were compared, unique heterochromatic polymorphisms were detected on chromosome pairs 1 and 5. Specifically, specimens had heterochromatic blocks (h+h+) on both chromosomes, one chromosome with heterochromatic blocks (h+h-) or chromosomes that lacked heterochromatic blocks (h-h-). Considering that heteromorphic pattern is not correlated with variation in size, the process of heterochromatinization might act on the long arms of these chromosomes. In summary, all chromosomal markers indicate that the karyotype of *Hypostomus iheringii* is highly differentiated and that the heterochromatinization of chromosomal segments may have contributed to its karyotypic differentiation.

## Introduction

Loricariidae is a speciose group of Neotropical fishes that is composed of six subfamilies: Hypoptopomatinae, Hypostominae, Lithogeneinae, Loricariinae, Neoplecostominae, and Delturinae (Armbruster 2004, Reis et al. 2006, Cramer et al. 2011). Armbruster (2004) considered the old subfamily Ancistrinae to be a synonym of Hypostominae, a group that currently consists of the tribes Corymbophanini, Rhinelepini, Hypostomini, Pterygoplichthini and Ancistrini. *Hypostomus* is the type genus of Hypostominae and has great morphological (Weber 2003) and cytogenetic (Bueno et al. 2011) diversity. According to Weber (2003), the genus consists of a large number of species that exhibit a high level of morphological and color pattern variation, thus making systematic identification difficult. Of the more than 120 species that have been described within this group (Zawadzki et al. 2010), 21 were reported to reside in the Alto Paraná basin (Weber 2003, Jerep et al. 2007). Ziemniczak et al. (in press) concluded that the karyotypic differentiation of Hypostomini is correlated with the great diversification of form in this tribe and may have been important for genetic and reproductive isolation between species.

Cytogenetic studies in *Hypostomus* indicate that there is great variability in various karyotypic aspects, which contributes to enormous complexity of the group (Artoni and Bertollo 1996). The same chromosomal variations, such as karyotypic formula (Michele et al. 1977, Artoni and Bertollo 1996, Alves et al. 2006, Bueno et al. 2011), heterochromatin distribution (Artoni and Bertollo 1999, Rubert et al. 2008) and nucleolus organizer regions (Artoni and Bertollo 2001, Rubert et al. 2008), occur frequently within populations. However, population polymorphisms are rare and manifest as variations in the karyotypic formula (Artoni and Bertollo 1999).

The amplification and mobility of heterochromatic blocks of chromosomes are well documented in some organisms (Hamilton et al. 1990, Modi 1993). Sequences of satellite DNA appear to play an important role in the evolution of the genome of different organisms by promoting chromosome rearrangements and exhibiting rapid differentiation due to intragenomic mobility taking important role in karyotype evolution and speciation due to gene flow restriction (Wichman et al. 1991, Kantek et al. 2009, Machado et al. 2011).

In this study, the first karyotype analysis of *Hypostomus iheringii* (Regan, 1908) was performed using classic (Giemsa staining, C-banding, and Ag-NOR) and molecular (fluorescence *in situ* hybridization - FISH) cytogenetic methods, emphasizing the distribution of heterochromatic blocks, interrelating and discussing the possible role of heterochromatin in the diversification of the genomes of Loricariidae.

## Materials and methods

### Animals and mitotic chromosome preparations

Nine specimens (five males and four females) of *Hypostomus iheringii* from the Córrego da Lapa, a tributary of the Passa-Cinco River in Ipeúna, São Paulo, Brazil, were analyzed. These specimens were deposited in the Museum of Zoology at the University of São Paulo, under voucher number MZUSP 106769. The animals were anesthetized with clove oil, according to the method described by Griffiths (2000), and then sacrificed. The procedures were performed following the guidelines of the Committee of Ethics in Animal Experimentation (Process CEUA 07/2011) at Universidade Estadual de Ponta Grossa. Cell suspensions containing mitotic chromosomes were obtained from the cells of the anterior portion of the kidney of these specimens according to the procedures described by Bertollo et al. (1978) and Foresti et al. (1993).

### Chromosome staining

The chromosomes were stained with a solution of 5% Giemsa. C-banding was performed following the protocol described by Sumner (1972) with modifications in staining method (Lui et al. 2009). The nucleolus organizer regions (Ag-NORs) were determined according to the method described by Howell and Black (1980). These methods were performed sequentially (conventional staining of the chromosomes with Giemsa, C-banding and Ag-NORs) for accuracy in identifying chromosome pairs.

### Chromosome hybridization, probes and karyotype analysis

The physical mapping of 18S and 5S rDNA on the chromosomes was obtained by FISH, as described by Pinkel et al. (1986), with probes obtained from *Prochilodus argenteus* Spix and Agassiz, 1829 (Hatanaka and Galetti Jr. 2004) and *Leporinus elongates* Valenciennes, 1850 (Martins and Galetti Jr. 1999). The probes of 5S and 18S rDNA were labeled with digoxigenin-11-dUTP and biotin-14-dATP, respectively, by *nick translation*, according to the manufacturer’s instructions (Roche Applied Science). The hybridization procedure was performed under high stringency conditions (2.5 ng/µL of each probe, 50% deionized formamide, 10% dextran sulphate, 2XSSC, pH 7.0–7.2, at 37°C overnight). After hybridization, the slides were washed in 15% formamide/0.2XSSC at 42°C for 20 min, 0.1XSSC at 60°C for 15 min and 4XSSC/0.05% Tween at room temperature for 10 min. The last step was performed in two 5 min washes. The signal detection was performed using streptavidin-Alexa Fluor 488 (Molecular Probes) against the 18S rDNA and anti-digoxigenin-rhodamine (Roche Applied Science) for the 5S rDNA probes. The chromosomes were counter-stained with a solution of antifading/DAPI (40 µL of antifading + 1 µL of DAPI – 0.2 mg/mL) and analyzed under an Olympus BX50 epifluorescence microscope.

The chromosomes were classified as metacentric (m), submetacentric (sm), subtelocentric (st), and acrocentric (a), according to the ratio of arms proposed by Levan et al. (1964), and arranged to form the karyotype in descending order by size. The software Image-Pro Plus 6.3 (Media Cybernetics) was used for image capture.

## Results

**Figure 1.**
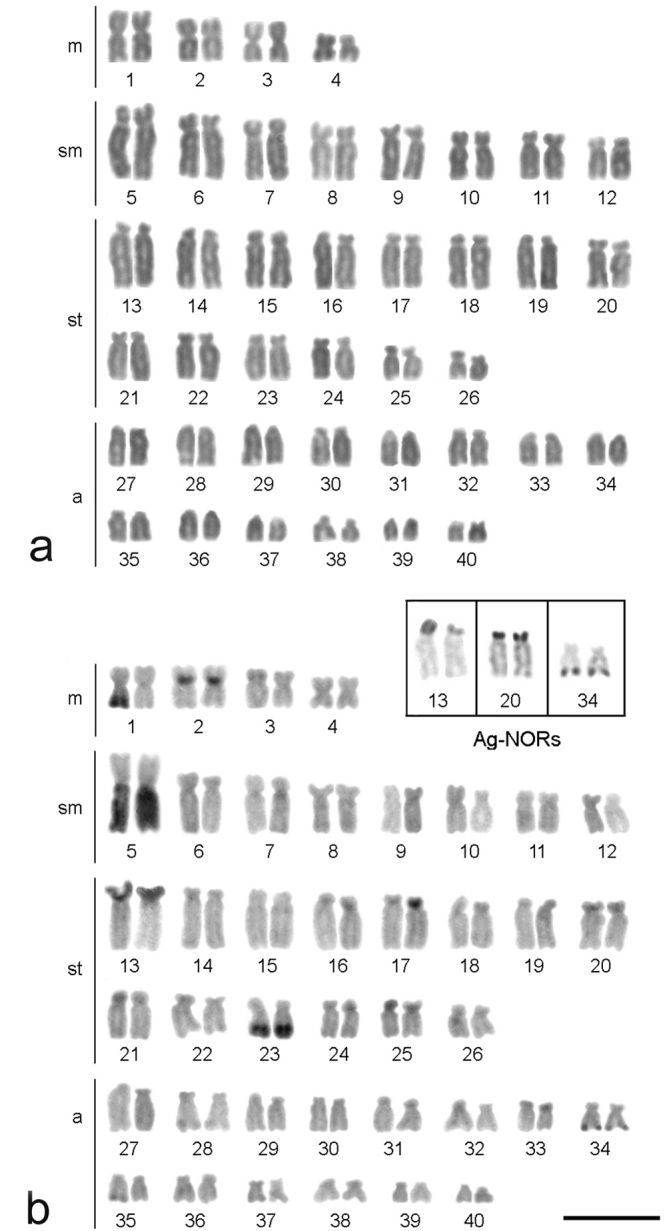
Karyotypes of female *Hypostomus iheringii* arranged from Giemsa-stained (**a**) and C-banded chromosomes (**b**). The chromosome pairs carrying Ag-NORs are boxed. Bar = 5 µm.

**Figure 2.**
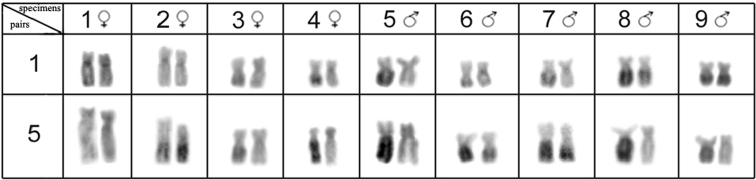
Accentuated heterochromatic polymorphisms on chromosome pairs 1 and 5 of the *Hypostomus iheringii*.

**Figure 3.**
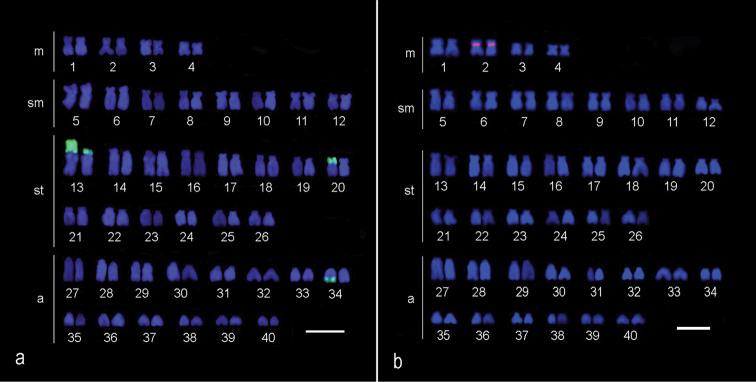
Karyotype of female *Hypostomus iheringii* submitted to FISH using 18S rDNA probe (**a**) and submitted to FISH using 5S rDNA probe (**b**). Bars = 5 µm.

All of the *Hypostomus iheringii* specimens showed a diploid number of 80 chromosomes (8m + 16sm + 28st + 28a, NF=132) without a heteromorphic sexual system (Fig. 1a). C-banding analysis revealed a small section of heterochromatin, preferentially located in the terminal portions of some of the chromosomes (Fig. 1b). Two conspicuous heterochromatic blocks were found in the terminal position of the long arm of chromosome pairs 1 (m) and 5 (sm) with inter-individual variations. Specifically, specimens had heterochromatic blocks (h+h+) on both chromosomes, one chromosome with heterochromatic blocks (h+h-) or chromosomes that lacked heterochromatic blocks (h-h-) (Fig. 2). Silver nitrate staining revealed the presence of multiple nucleolus organizer regions (NORs) located in the terminal portion of the short arm of two pairs of subtelocentric chromosomes (pairs 13 and 20) and in the terminal position of the long arm of an acrocentric chromosome pair (chromosome 34) (Fig. 1, in Box). In addition, a size heteromorphism was found in the Ag-NORs sites of chromosome pair 13 in all individuals analyzed. FISH with the 18S rDNA probe confirmed the silver nitrate staining result for pair 13, but only one of the homologues of the pairs 20 and 34 was marked (Fig. 3a). FISH revealed that the 5S rDNA sites were located in the interstitial portion of the short arm of a metacentric chromosome (pair 2) (Fig. 3b).

## Discussion

Cytogenetic studies in Loricariidae reveal a remarkable diversity of chromosomal numbers, formulae and markers. Despite this extensive variation, karyotype analyses of the species in this family have allowed well-defined evolutionary trends and putative group relationships to be inferred (Artoni and Bertollo 2001, Alves et al. 2006, Ziemniczak et al. in press). In *Hypostomus*, the diploid number ranges from 54 chromosomes in *Hypostomus plecostomus* Linnaeus, 1758 (Muramoto et al. 1968) to 84 chromosomes in *Hypostomus* sp. 2 (Cereali et al. 2008). Chromosome sets that are numerically similar to *Hypostomus* sp. 2 were found in *Hypostomus* sp. 3 from the Salobrinha Stream in Mato Grosso do Sul, Brazil (Cereali et al. 2008) (82 chromosomes), *Hypostomus* sp. E from the Mogi-Guaçu River in São Paulo, Brazil (Artoni and Bertollo 1996) (80 chromosomes) and *Hypostomus topavae* (Godoy, 1969) from the Piquiri River in Paraná, Brazil (Bueno et al. 2011) (80 chromosomes). Considering these data, *Hypostomus iheringii* (2n = 80) (Fig. 1a) is among the species with the highest chromosome number in the *Hypostomus*. In Loricariidae, the diploid number ranges from 2n=34 chromosomes in *Ancistrus cuiabae* Knaack, 1999 (Mariotto et al. 2011) to 2n=96 chromosomes in *Upsilodus* sp. (Kavalco et al. 2004). The plesiomorphic state is considered to be 2n=54 chromosomes (Artoni and Bertollo 2001). According to Artoni and Bertollo (2001), species of *Hypostomus* with a higher chromosome number are likely to be more derived than species with 2n=54 chromosomes. This hypothesis is supported by the observation that 2n=54 chromosomes is a condition that is shared with the outgroup Trichomycteridae (Ziemniczak et al. in press) and is visualized in all Loricariidae subfamilies as well. Thus, because *Hypostomus iheringii* has a high chromosome number, we conclude that it may represent a derived species in this genus.

The increase in the number of st/a chromosomes was postulated to be directly proportional to 2n while the number of m/sm chromosomes is inversely proportional to 2n. This hypothesis would suggest that centric fissions have played a key role in karyotype evolution of this group (Artoni and Bertollo 2001). Recently, the work of Bueno et al. (2011) supported this hypothesis only for species with chromosome numbers higher than or equal to 80 chromosomes. For species with lower chromosome numbers, however, it was not possible to correlate the diploid number of chromosomes with the proportion of st/a chromosomes. Then, other chromosomal rearrangements, such as inversions, deletions, duplications and heterochromatiniztion, could contribute to the chromosomal differentiation of tribe Hypostomini.

Polymorphisms of heterochromatic blocks with maintenance of heteromorphic states are relatively common among Teleost fishes and are correlated to population differentiation and speciation (Hartley and Horne 1984, Mantovani et al. 2000, Vicari et al. 2003, Souza et al. 2007, Kantek et al. 2009, Bellafronte et al. 2011). In Loricariidae, population variation in the number and size of heterochromatic sites was described for *Hisonotus leucofrenatus* Miranda Ribeiro, 1908 (Andreata et al. 2010), *Kronichthys lacerta* Nichols, 1919 and *Isbrueckerichthys duseni* Miranda Ribeiro, 1907 (Ziemniczak et al. in press). This form of variation in *Hypostomus* has been found for *Hypostomus iheringii* in this study (Fig. 2) and in a previous report for *Hypostomus* sp. B (Artoni and Bertollo 1999), where an extra chromosome with a completely heterochromatic arm was observed in two specimens.

In *Hypostomus iheringii*, the polymorphism of the heterochromatic regions in chromosome pairs is not correlated to size variations in these euchromatic chromosomes (Fig. 2). Therefore, the process of heterochromatinization (inactivation by conversion of euchromatin into heterochromatin) might act on the long arms of chromosomes 1 and 5. However, the occurrence of additional amplification in these heterochromatic chromosomal regions cannot be ruled out completely.

Physical mapping of the 45S rDNA multigene family revealed a lack of staining of one homologue of chromosome pairs 20 and 34 (Fig. 3), probably because of unequal crossing over between homologues of these pairs resulting in sites of different sizes (Markovic et al. 1978). Accordingly, FISH failed to detect such sites as a result of its limited ability to detect very small sizes (Schwarzacher and Heslop-Harrison 2000). According to Kavalco et al. (2005), a population of *Hypostomus affinis* (Steindachner, 1877) from Paraíba do Sul river Basin has multiple sites of 18S rDNA, but marking by FISH does not occur in all Ag-NOR positive chromosomes. Thus, the occurrence of small 18S rDNA sites in *Hypostomus* is possible. However, in *Hypostomus affinis*, the unequal crossing over was more conspicuous, leading to the emergence of an obvious size heteromorphism among homologous chromosomes. Size differences among NOR sites in this genus are more frequent, and several cases describe variations in the size of sites detectable by silver nitrate staining, similar to what was observed for pair 13 (Fig. 1, Box) (Artoni and Bertollo 1996, Kavalco et al. 2004, 2005, Cereali et al. 2008, Rubert et al. 2008).

Although the literature on the physical mapping of 5S rDNA in *Hypostomus* is not abundant, variations within the group for this marker have been reported. This study identified only one chromosome pair carrying these sites, pair 2 (Fig. 3); however, eight chromosomes bearing such sequences were identified for *Hypostomus affinis* (Kavalco et al. 2005), and nine chromosomes were identified for *Hypostomus regani* Ihering, 1905 (Mendes-Neto et al. 2011). The data available for this marker in other genera of Loricariidae indicate that the group has become quite diverse, with some species having a 5S rDNA simple mark, such as *Neoplecostomus micropis* (Steindachner, 1877) and *Harttia loricariformis* Steindachner, 1877 (Kavalco et al. 2004), and others having multiple, such as *Harttia carvalhoi* Miranda Ribeiro, 1939 (Centofante et al. 2006) and *Upsilodus* sp. (Kavalco et al. 2004). In species from the basal groups of Loricariidae, including *Kronichthys lacerta*, *Isbrueckerichthys duseni*, *Parotocinclus maculicauda* Steindachner, 1877, and the outgroup *Trichomycterus*, the synteny of the major and minor rDNAs has been detected and correlated to the primitive state (Ziemniczak et al. in press). The wide range of diploid number and relocation of the rDNAs in the karyotype of *Hypostomus affinis* represent a derived state.

In summary, *Hypostomus iheringii* displays evolutionary trends that are characteristic of the genus *Hypostomus*, such as the high number of subtelocentric and acrocentric chromosomes assigned to the species of this genus with high chromosome numbers. However, the distribution and diversification of heterochromatin suggests new evolutionary trends. All chromosomal markers indicate that the karyotype of *Hypostomus iheringii* is highly differentiated and that heterochromatinization of chromosomal segments may contribute to karyotypic differentiation found in this *Hypostomus iheringii* population.
